# Factors Associated With Opioid Use in Patients Hospitalized for Acute Pancreatitis

**DOI:** 10.1001/jamanetworkopen.2019.1827

**Published:** 2019-04-12

**Authors:** Bechien U. Wu, Rebecca K. Butler, Wansu Chen

**Affiliations:** 1 Center for Pancreatic Care, Division of Gastroenterology, Kaiser Permanente Los Angeles Medical Center, Los Angeles, California; 2 Department of Research and Evaluation, Kaiser Permanente Southern California, Pasadena

## Abstract

**Importance:**

Limited guidance exists regarding the optimal approach to management of pain in acute pancreatitis (AP).

**Objectives:**

To investigate sources of variability in opioid use for treatment of acute pain in patients hospitalized for AP and to explore a potential association of opioid prescribing patterns with length of stay.

**Design, Setting, and Participants:**

This retrospective cohort study included 4307 patients 18 years and older hospitalized for AP in a community-based integrated health care system, from January 1, 2008, to June 30, 2015. Analysis began in November 2017.

**Exposures:**

Opioid use was quantified by morphine equivalent dose (MED).

**Main Outcomes and Measures:**

Three analyses were performed: (1) factors associated with increased opioid administration during the initial 12 hours of hospitalization (baseline), (2) association of baseline opioid use with length of stay, and (3) frequency of opioid use 90 days after hospital discharge (persistent use).

**Results:**

The cohort included 4307 patients (median [interquartile range] age, 57.4 [44.0-70.2] years; 2241 women [52.0%]) with AP. At baseline, 3443 patients (79.9%) received opioids, and 388 patients (9.6%) had persistent opioid use after discharge. After adjusting for pain and other clinical factors, women received less MED than men (adjusted event ratio, 0.83; 95% CI, 0.79-0.86; *P* < .001). Hispanic and Asian patients received less MED than non-Hispanic white patients (adjusted event ratio, 0.85; 95% CI, 0.81-0.90; *P* < .001; and adjusted event ratio, 0.79; 95% CI, 0.72-0.86; *P* < .001, respectively). Alcohol-related AP etiology was associated with increased MED vs gallstone disorders (adjusted event ratio, 1.11; 95% CI, 1.05-1.18; *P* < .001). Two of 13 hospitals administered significantly less opioids compared with the others. Median (interquartile range) length of stay was independently associated with MED at baseline, with 3.0 (2.1-4.5) days among patients not receiving opioids vs 5.0 (3.2-8.7) days among patients in the highest quintile of MED (*P* < .001).

**Conclusions and Relevance:**

In addition to pain and disease severity, opioid use varied by etiology of AP, sex, race/ethnicity, and institution of treatment. Increased opioid use at baseline was associated with longer hospitalization. These findings suggest opportunities for improved approaches to pain control for patients with AP.

## Key Points

**Question:**

What factors are associated with increased opioid use in patients hospitalized for acute pancreatitis?

**Findings:**

In this cohort study of 4307 patients hospitalized for acute pancreatitis in a US integrated care system, 3443 patients (79.9%) received initial treatment with opioids. Younger patient age, male sex, white race, non-Hispanic ethnicity, and alcohol-related etiology of pancreatitis were independently associated with increased opioid use after adjusting for pain and disease activity.

**Meaning:**

These findings suggest opportunities for improved approaches to control pain in acute pancreatitis.

## Teaser

This cohort study investigates the variability in opioid use in pain management for patients hospitalized for acute pancreatitis.

## Introduction

Acute pancreatitis (AP) is one of the most common reasons for hospitalization related to a digestive illness in the United States, accounting for more than 250 000 hospitalizations on an annual basis,^1,2^ with an incidence of 111 per 100 000 persons.^3^ The cornerstones of treatment include supportive measures, such as bowel rest, intravenous fluid hydration, and analgesia. While previous studies and 2018 guidelines^4^ have begun to critically assess the roles of food restriction and aggressive fluid resuscitation, limited data exist regarding optimal approaches to control pain in the routine care of patients with AP.

In the United States, opioids have been the mainstay of treatment for pain control for patients with AP. However, to our knowledge, limited guidance exists regarding the optimal use of these medications. Specifically, current clinical practice guidelines from the American Gastroenterological Association,^4^ the American College of Gastroenterology,^5^ and the International Association of Pancreatology in collaboration with the American Pancreatic Association^6^ do not comment on approaches to acute pain management for patients with AP. Considering the increasing recognition of harms associated with opioid use,^7,8,9^ greater insight into current prescribing patterns and assessment of the potential impact of these agents on disease course are urgently needed. Based on the lack of clear guidelines on the use of opioids in this setting, we hypothesized that prescribing patterns for these medications would vary considerably among patients with AP. We further sought to explore any potential association of opioid use with length of hospitalization.

The objective of this study was to conduct a broad assessment of opioid use in the treatment of patients with AP during and after hospitalization within a multihospital, community-based integrated health care system. Specifically, our study aims were to assess institutional and patient characteristics associated with increased use of opioids during initial treatment and the potential association of early opioid use with duration of hospital stay and with prolonged opioid use after patients were discharged from the hospital.

## Methods

### Study Design and Setting

**Figure 1.**
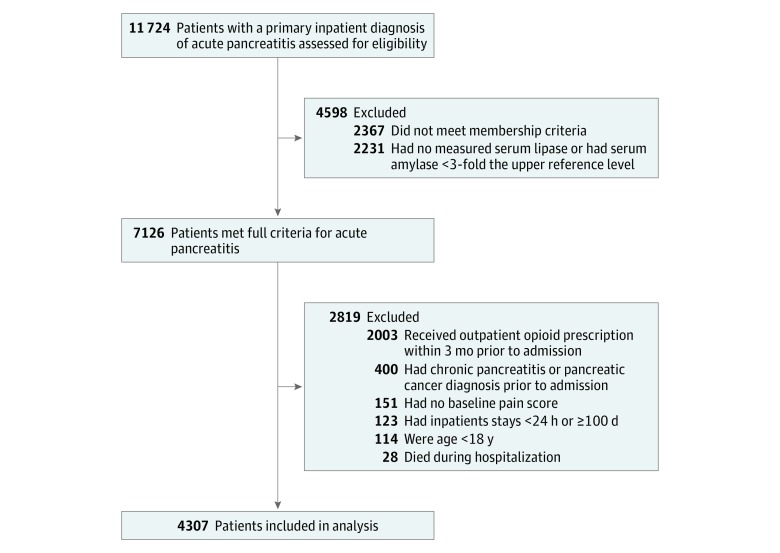
Flow Diagram of Study Cohort

We conducted a retrospective observational cohort study including patients hospitalized for a principle diagnosis of AP in Kaiser Permanente Southern California from January 1, 2008, to June 30, 2015. Kaiser Permanente is an integrated health care system that comprises 9 regions, of which Southern California is the largest, with 14 acute care hospitals that provide health care services for a population of more than 4.6 million active health plan enrollees. The health plan enrollees receive comprehensive services encompassing the full spectrum of care, including ambulatory, inpatient, laboratory, and pharmacy. This study was approved by the Kaiser Permanente Southern California Institutional Review Board, and informed consent was waived, given the retrospective nature of the study that involved no direct patient contact. This study is reported in accordance with the Strengthening the Reporting of Observational Studies in Epidemiology (STROBE) reporting guideline (Figure 1). Data analysis began November 2017.

### Study Population

Adult patients (aged ≥18 years) with AP were identified through a combination of discharge diagnosis codes (*International Classification of Diseases, Ninth Revision* code 577.1) and an elevated serum amylase or serum lipase level (≥3-fold upper limit of normal) during hospitalization. This definition was used previously and showed a positive predictive value of more than 95% compared with manual medical record validation based on clinical criteria.^10^ The first hospitalization that met the inclusion criteria was identified as the index hospitalization, and the first 12 hours during the index hospitalization was defined as the baseline period. We excluded 2819 patients (1) with a history of chronic pancreatitis, (2) with outpatient opioids dispensed within 90 days prior to the index hospitalization, (3) with an index hospital stay less than 24 hours or longer than 100 days, (4) without a recorded pain score at baseline, or (5) who died during the index hospitalization. Patients were further required to have at least 1 year of membership prior to index hospitalization to ensure adequate assessment for comorbid illnesses. Data from all patients satisfying the study criteria were incorporated into the primary analysis. Only patients with at least 180 days’ continuous membership after discharge were included in subsequent analysis of opioid use following hospitalization.

### Variable Definitions

We focused on the initial 12 hours of hospitalization as a measure of opioid use, given the potential variability in pain symptoms and disease course during subsequent stages of hospitalization. We quantified the baseline opioid use as intravenous morphine equivalent doses (MEDs) of narcotic analgesic medication dispensed at specific times during hospitalization. Baseline opioid dispensation was calculated as the sum of MEDs dispensed during the initial 12 hours of hospitalization. *Opioid use at discharge* was defined as dispensation of any opioid prescription within 14 days after discharge from the index hospitalization. *Persistent opioid use* was defined as opioid dispensation at discharge or within the first 2 weeks after discharge and again within 90 to 180 days from the discharge date of the index hospitalization. This period was selected based on 2017 surgical literature evaluating persistent opioid use after surgical procedures^11^ and is consistent with definitions set forth by the International Association for the Study of Pain.^12^

Pain at baseline was assessed as the maximum recorded pain score on a numeric rating scale of 0 to 10. Numeric pain scores were abstracted from electronic medical records and were typically assessed at regular intervals, along with additional vital sign measurements, in the context of routine clinical care. Disease activity at baseline was evaluated based on the presence of systemic inflammatory response syndrome (SIRS)^13^ and number of SIRS criteria present (range, 0-4) at baseline.

Additional study variables included age, sex, self-reported race/ethnicity, neighborhood median household income, percentage of residents with less than high school education in the neighborhood, Charlson Comorbidity Index score,^14^ alcohol use disorder (ever, never, or unknown), smoking (ever, never, or unknown), body mass index (calculated as weight in kilograms divided by height in meters squared), etiologies of AP (alcohol use disorder, either self-reported at the time of outpatient visits or any reported history of alcohol use disorder in the year before index hospitalization; gallstone disease according to *International Classification of Diseases, Ninth Revision* code 574.x in the year before or year after index hospitalization; or cholecystectomy [*Current Procedural Terminology* codes 47562, 47563, 47600, and 47605] in the 180 days after index hospitalization), maximum pain score during the baseline, medical center where the patient was admitted for index hospitalization, and length of stay (LOS) during the index hospitalization. The 2 neighborhood-level variables were derived from members’ addresses using block group–level estimates provided by Claritas.

### Statistical Analysis

#### Baseline Opioid Use

We attempted to understand the prescribing patterns for opioid use at baseline. Patient demographic characteristics and clinical and institutional characteristics were compared among patients with levels of baseline opioid use characterized by quintile (0, 1-4, 5-8, 9-14, and ≥15 MED) by using the χ^2^ test for categorical variables and Kruskal-Wallis test for continuous variables. The mean baseline opioid use in MED, with 95% CIs, was calculated by patient demographic and clinical characteristics, including age, sex, race/ethnicity, maximum recorded pain level at baseline, number of SIRS criteria, Charlson Comorbidity Index score, etiology of AP, and medical center (an institutional factor). We then performed multivariable Poisson regression with a robust error variance to assess the association of these parameters with opioid use during the baseline period of hospitalization. The ratio of the expected number of MED between any 2 groups being compared (event ratio) and the 95% CIs were reported for each factor.

#### Baseline Opioid Use and LOS

We evaluated the association of baseline opioid use with LOS using multivariable simple linear regression. We used log-transformed LOS, adjusting for the associations of patient, clinical, and institutional characteristics with outcomes (presented in percentage changes in LOS of 2 comparison groups and 95% CIs).

#### Persistent Opioid Use Following Hospital Discharge

We sought to assess the patterns of opioid dispensation at and after discharge and factors associated with new persistent opioid use. Patient characteristics, including MED at baseline, total MED during the entire hospital stay, and MED per day, were compared among 3 groups of patients based on opioid dispensing status at or after discharge: (1) opioids were dispensed neither at discharge nor within 14 days after discharge, (2) opioids were dispensed either at discharge or within 14 days after discharge, and (3) opioids were dispensed at discharge or within 14 days after discharge and again at least once within 90 to 180 days after discharge. The groups were compared using the χ^2^ test for categorical variables and Kruskal-Wallis test for continuous variables. A multivariable logistic regression model was applied to assess the association of baseline MED and mean daily MED during the entire hospital stay with risk of persistent opioid use, after controlling for the potential risk factors. In this stage of the analysis, patients who died, terminated the enrollment from the Kaiser Permanente Southern California health plan, or lost prescription drug benefits within 180 days after discharge were excluded.

Analyses were performed in SAS statistical software version 9.3 (SAS Institute). Reported *P* values are 2-sided, and the level of significance was set at α = .05.

## Results

We identified 4307 patients (median [interquartile range] age, 57.4 [44.0-70.2] years; 2241 women [52.0%]) with AP who met study inclusion criteria (Figure 1). Of these, 3443 patients (79.9%) received opioids during the initial 12 hours of hospitalization.

### Pattern of Opioid Use at Baseline

Baseline demographic and clinical characteristics for the study cohort are presented in Table 1. The study cohort was racially/ethnically diverse, comprising 1802 Hispanic patients (41.8%), 1693 non-Hispanic white patients (39.3%), 413 non-Hispanic black patients (9.6%), and 351 non-Hispanic Asian patients (8.1%). A total of 2354 patients (54.7%) had evidence of gallstone-related diseases, while 1471 patients (34.2%) had a history of alcohol use. Older age, being a woman, being non-Hispanic Asian, having gallstone disorders, and having a higher Charlson Comorbidity Index score were negatively associated with the amount of prescribed opioids at baseline, while alcohol use disorder history, smoking history, and pain score were positively associated with the amount of opioid used (Table 1).

**Table 1.** Patient Demographic and Clinical Characteristics by Opioid Use in MED at Baseline^a^

Characteristic	No. (%)						*P* Value
	0 MED (n = 864)	1-4 MED (n = 997)	5-8 MED (n = 879)	9-14 MED (n = 769)	≥15 MED (n = 798)	Total (N = 4307)	
Age, median (IQR), y	65.7 (50.8-77.9)	60.7 (46.9-74.1)	57.2 (43.8-69.8)	54.5 (40.7-64.5)	49.9 (39.4-60.5)	57.4 (44.0-70.2)	<.001
Female	468 (54.2)	584 (58.6)	498 (56.7)	387 (50.3)	304 (38.1)	2241 (52.0)	<.001
Race/ethnicity							
Asian, non-Hispanic	87 (10.1)	94 (9.4)	72 (8.2)	54 (7.0)	44 (5.5)	351 (8.1)	<.001
Black, non-Hispanic	73 (8.4)	108 (10.8)	86 (9.8)	73 (9.5)	73 (9.1)	413 (9.6)	
Hispanic	322 (37.3)	426 (42.7)	404 (46.0)	338 (44.0)	312 (39.1)	1802 (41.8)	
Other, non-Hispanic	14 (1.6)	8 (0.8)	8 (0.9)	8 (1.0)	10 (1.3)	48 (1.1)	
White, non-Hispanic	368 (42.6)	361 (36.2)	309 (35.2)	296 (38.5)	359 (45.0)	1693 (39.3)	
Neighborhood income, median (IQR), $ in thousands	60.1 (45.6-80.5)	57.5 (43.0-77.1)	61.1 (43.8-79.3)	61.6 (45.0-81.3)	62.1 (46.4-82.0)	60.5 (44.5-79.8)	.007
BMI, mean (IQR)	27.1 (23.6-31.1)	28.5 (24.8-32.8)	29.1 (25.5-33.4)	29.9 (26.3-35.0)	29.8 (26.4-34.5)	28.8 (25.1-33.3)	<.001
Charlson Comorbidity Index score							
0	320 (37.0)	382 (38.3)	385 (43.8)	359 (46.7)	381 (47.7)	1827 (42.4)	<.001
1-2	315 (36.5)	356 (35.7)	309 (35.2)	254 (33.0)	265 (33.2)	1499 (34.8)	
≥3	201 (23.3)	197 (19.8)	133 (15.1)	96 (12.5)	75 (9.4)	702 (16.3)	
Missing	28 (3.2)	62 (6.2)	52 (5.9)	60 (7.8)	77 (9.6)	279 (6.5)	
Smoking history							
Current	64 (7.4)	65 (6.5)	93 (10.6)	86 (11.2)	108 (13.5)	416 (9.7)	<.001
Former	216 (25.0)	235 (23.6)	183 (20.8)	166 (21.6)	181 (22.7)	981 (22.8)	
Never	95 (11.0)	119 (11.9)	81 (9.2)	99 (12.9)	131 (16.4)	525 (12.2)	
Missing or unknown	489 (56.6)	578 (58.0)	522 (59.4)	418 (54.4)	378 (47.4)	2385 (55.4)	
Alcohol use disorder history							
Ever	261 (30.2)	299 (30.0)	281 (32.0)	305 (39.7)	325 (40.7)	1471 (34.2)	<.001
Never	480 (55.6)	551 (55.3)	478 (54.4)	357 (46.4)	351 (44.0)	2217 (51.5)	
Unknown	123 (14.2)	147 (14.7)	120 (13.7)	107 (13.9)	122 (15.3)	619 (14.4)	
Etiology of AP							
Gallstone disorders	504 (58.3)	607 (60.9)	495 (56.3)	414 (53.8)	334 (41.9)	2354 (54.7)	<.001
Alcohol related	110 (12.7)	116 (11.6)	138 (15.7)	160 (20.8)	201 (25.2)	725 (16.8)	
Other	250 (28.9)	274 (27.5)	246 (28.0)	195 (25.4)	263 (33.0)	1228 (28.5)	
Maximum pain score at baseline							
0	334 (38.7)	61 (6.1)	14 (1.6)	5 (0.7)	0	414 (9.6)	<.001
1-3	172 (19.9)	71 (7.1)	21 (2.4)	1 (0.1)	2 (0.3)	267 (6.2)	
4-7	262 (30.3)	486 (48.7)	312 (35.5)	199 (25.9)	96 (12.0)	1355 (31.5)	
8-10	96 (11.1)	379 (38.0)	532 (60.5)	564 (73.3)	700 (87.7)	2271 (52.7)	
Medical center^b^							
A	81 (9.4)	109 (10.9)	88 (10.0)	77 (10.0)	69 (8.6)	424 (9.8)	<.001
B	77 (8.9)	93 (9.3)	94 (10.7)	93 (12.1)	74 (9.3)	431 (10.0)	
C	66 (7.6)	71 (7.1)	80 (9.1)	71 (9.2)	60 (7.5)	348 (8.1)	
D	80 (9.3)	84 (8.4)	74 (8.4)	53 (6.9)	40 (5.0)	331 (7.7)	
E	8 (0.9)	23 (2.3)	14 (1.6)	14 (1.8)	14 (1.8)	73 (1.7)	
F	57 (6.6)	55 (5.5)	54 (6.1)	56 (7.3)	45 (5.6)	267 (6.2)	
G	44 (5.1)	49 (4.9)	51 (5.8)	42 (5.5)	35 (4.4)	221 (5.1)	
H	17 (2.0)	18 (1.8)	20 (2.3)	17 (2.2)	25 (3.1)	97 (2.3)	
I	39 (4.5)	69 (6.9)	67 (7.6)	57 (7.4)	84 (10.5)	316 (7.3)	
J	78 (9.0)	68 (6.8)	52 (5.9)	42 (5.5)	71 (8.9)	311 (7.2)	
K	112 (13.0)	150 (15.0)	133 (15.1)	110 (14.3)	133 (16.7)	638 (14.8)	
L	62 (7.2)	72 (7.2)	49 (5.6)	63 (8.2)	56 (7.0)	302 (7.0)	
M	58 (6.7)	61 (6.1)	51 (5.8)	31 (4.0)	42 (5.3)	243 (5.6)	
N	85 (9.8)	75 (7.5)	52 (5.9)	43 (5.6)	50 (6.3)	305 (7.1)	

Abbreviations: AP, acute pancreatitis; BMI, body mass index (calculated as weight in kilograms divided by height in meters squared); IQR, interquartile range; MED, morphine equivalent dose.

^a^ Baseline period is defined as the first 12 hours after the admission of index hospitalization for acute pancreatitis.

^b^ Medical centers are identified by anonymized letters.

**Figure 2.**
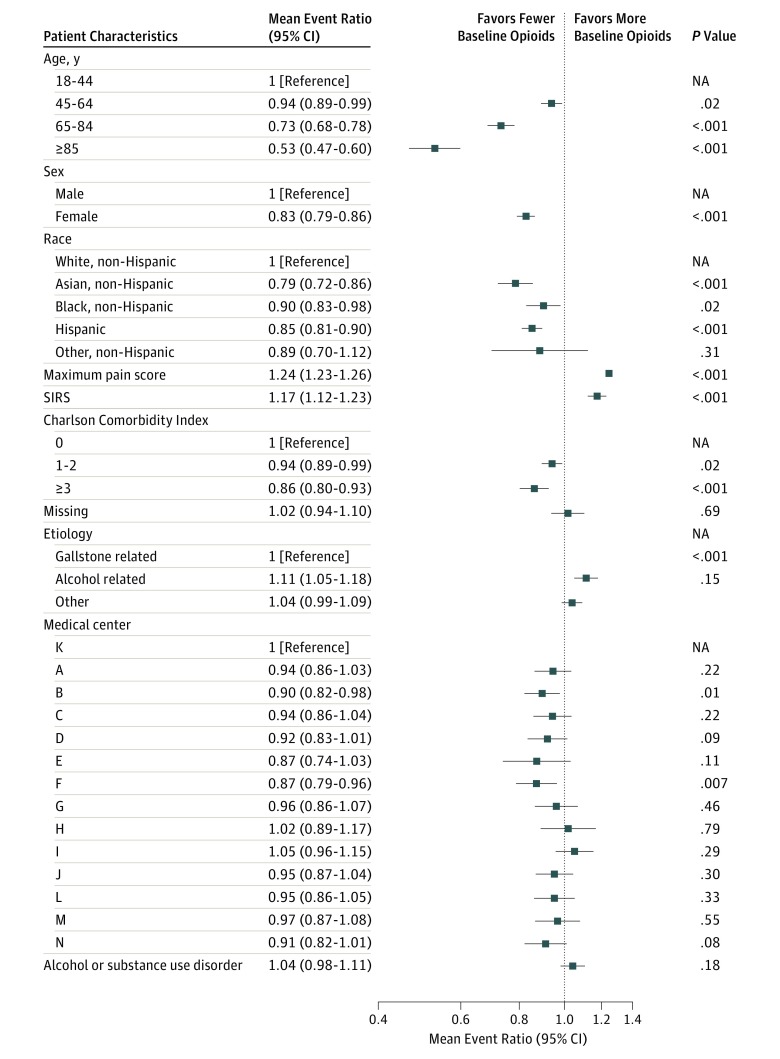
Event Ratio of Expected Morphine Equivalent Dose and 95% CI NA indicates not applicable; SIRS, systemic inflammatory response syndrome.

Opioid administration varied based on patient age, sex, race/ethnicity, pain level, Charlson Comorbid Index score, etiology of AP, mental health condition, and medical center (Table 2). The median MED in the youngest age group (age 18-44 years) was 4-fold larger than the oldest age group (≥85 years). The median MED at baseline varied from 0 in patients without pain to 10 among those whose maximum pain score was 8 to 10. The median MED also seemed to increase with the number of SIRS criteria met. Patients whose AP etiology was alcohol related seemed to have higher MEDs compared with patients whose AP etiology was related to gallstone disorders. After adjusting for variables listed in Table 2 and prior substance use disorder history, evidence remained of significant variation in opioid administration based on patient age, sex, race/ethnicity, maximum pain score, disease activity level (SIRS criteria), Charlson Comorbidity Index score, etiology of AP, and medical center (Figure 2). Specifically, after adjusting for pain and other factors, women on average received 17% less opioids compared with men (adjusted event ratio, 0.83; 95% CI, 0.79-0.86; *P* < .001). In addition, Hispanic and non-Hispanic Asian patients received 15% and 21% less opiates, respectively, compared with non-Hispanic white patients (Hispanic vs non-Hispanic white: adjusted event ratio, 0.85; 95% CI, 0.81-0.90; *P* < .001; Asian vs non-Hispanic white: adjusted event ratio, 0.79; 95% CI, 0.72-0.86; *P* < .001). Alcohol-related AP etiology was associated with an 11% increase in opioid use at baseline compared with gallstone-related AP etiology (adjusted event ratio, 1.11; 95% CI, 1.05-1.18; *P* < .001). While controlling for patient-related and clinical factors removed much of the observed variation in opioid administration across medical centers, 2 of the 13 medical centers administered 10% to 13% less opiates at baseline compared with the reference hospital.

**Table 2.** Baseline^a^ Patient Characteristics and Opioid Use in MED

Characteristic	No. (N = 4307) (%)	MED, Median (Range) [IQR]
Age, y		
18-44	1156 (26.8)	8 (0-68) [4-16]
45-64	1664 (38.6)	8 (0-55) [4-14]
65-84	1259 (29.2)	4 (0-38) [0-8]
≥85	228 (5.3)	2 (0-24) [0-6]
Sex		
Male	2066 (48.0)	8 (0-68) [2-14]
Female	2241 (52.0)	6 (0-63) [2-10]
Race/ethnicity		
Asian, non-Hispanic	351 (8.1)	4 (0-36) [1-10]
Black, non-Hispanic	413 (9.6)	6 (0-49) [2-12]
Hispanic	1802 (41.8)	6 (0-63) [2-12]
Other, non-Hispanic	48 (1.1)	6 (0-48) [0-12]
White, non-Hispanic	1693 (39.3)	6 (0-68) [2-12]
Maximum pain score at baseline		
0	414 (9.6)	0 (0-14) [0]
1-3	267 (6.2)	0 (0-20) [0-4]
4-7	1355 (31.5)	4 (0-48) [2-8]
8-10	2271 (52.7)	10 (0-68) [6-16]
SIRS criteria met at baseline		
0	1524 (35.4)	4 (0-55) [0-10]
1	1462 (33.9)	6 (0-44) [2-12]
2	910 (21.1)	8 (0-52) [4-15]
3	382 (8.9)	10 (0-68) [4-18]
4	29 (0.7)	12 (0-36) [6-16]
Charlson Comorbidity Index score		
0	1827 (42.4)	8 (0-68) [3-13]
1-2	1499 (34.8)	6 (0-63) [2-12]
≥3	702 (16.3)	4 (0-44) [0-8]
Etiology of AP		
Gallstone related	2354 (54.7)	6 (0-52) [2-11]
Alcohol related	725 (16.8)	8 (0-68) [4-16]
Other	1228 (28.5)	6 (0-63) [2-12]
Medical center^b^		
A	424 (9.8)	6 (0-63) [2-12]
B	431 (10.0)	7 (0-48) [2-12]
C	348 (8.1)	7 (0-68) [2-12]
D	331 (7.7)	5 (0-52) [2-10]
E	73 (1.7)	7 (0-36) [4-12]
F	267 (6.2)	6 (0-36) [2-12]
G	221 (5.1)	7 (0-49) [2-12]
H	97 (2.3)	8 (0-40) [4-16]
I	316 (7.3)	8 (0-44) [4-16]
J	311 (7.2)	6 (0-52) [0-12]
K	638 (14.8)	7 (0-55) [3-12]
L	302 (7.0)	6 (0-48) [2-12]
M	243 (5.6)	6 (0-44) [2-12]
N	305 (7.1)	4 (0-46) [0-10]
Adjustment, anxiety, and mood disorders		
No	3410 (79.2)	6 (0-63) [2-12]
Yes	897 (20.8)	6 (0-68) [2-12]
Suicidality and intentional self-inflicted injury		
No	4282 (99.4)	6 (0-68) [2-12]
Yes	25 (0.6)	8 (0-36) [4-19]
Disruptive behavior disorders		
No	4286 (99.5)	6 (0-68) [2-12]
Yes	21 (0.5)	10 (0-30) [4-16]
Personality disorders		
No	4299 (99.8)	6 (0-68) [2-12]
Yes	8 (0.2)	5.5 (0-16) [0-12]
Schizophrenia and other psychotic disorders		
No	4270 (99.1)	6 (0-68) [2-12]
Yes	37 (0.9)	4 (0-36) [0-8]
Alcohol and other substance use disorders		
No	3687 (85.6)	6 (0-63) [2-12]
Yes	620 (14.4)	8 (0-68) [4-16]
Miscellaneous mental health disorders		
No	4199 (97.5)	6 (0-68) [2-12]
Yes	108 (2.5)	8 (0-36) [2-12]

Abbreviations: IQR, interquartile range; MED, morphine equivalent dose; SIRS, systemic inflammatory response syndrome.

^a^ Baseline period is defined as the first 12 hours after the admission of index hospitalization for acute pancreatitis.

^b^ Medical centers are identified by anonymized letters.

### Opioid Use at Baseline and Duration of Hospital Stay

Among other factors, increased use of MED at baseline was independently associated with a longer index hospital stay (Table 2). Median (interquartile range) length of stay was 3.0 (2.1-4.5) days among patients not receiving opioids vs 5.0 (3.2-8.7) days among patients in the highest quintile of MED (*P* < .001). After adjusting for age, sex, race/ethnicity, maximum pain score, baseline organ failure status, SIRS at baseline, Charlson Comorbidity Index score, etiology of AP, smoking history, drinking history, and medical center, the duration of stay increased by 2.4% (95% CI, 2.2%-2.7%; *P* < .001) for each unit increase of MED (Table 3).

**Table 3.** Factors Associated With Increased LOS

Characteristic	Crude LOS, Median (Range) [IQR], d	Adjusted Change, % (95% CI)	*P* Value
Age, y			
18-44	3.2 (1.0-89.9) [2.3-4.7]	1 [Reference]	NA
45-64	3.8 (1.0-97.9) [2.6-5.7]	10.9 (6.1 to 15.8)	<.001
65-84	4.0 (1.0-97.1) [2.7-6.2]	24.7 (18.4 to 31.4)	<.001
≥85	4.0 (1.1-59.0) [2.8-6.0]	35.4 (24.0 to 47.7)	<.001
Sex			
Male	3.7 (1.0-97.9) [2.5-5.9]	1 [Reference]	NA
Female	3.7 (1.0-83.9) [2.6-5.4]	0.5 (−3.1 to 4.2)	.79
Race/ethnicity			
White, non-Hispanic	3.7 (1.0-97.9) [2.6-5.7]	1 [Reference]	NA
Asian, non-Hispanic	3.9 (1.0-60.5) [2.6-6.2]	10.7 (3.5 to 18.4)	.003
Black, non-Hispanic	3.9 (1.0-78.2) [2.6-5.9]	6.7 (0 to 14.0)	.05
Hispanic	3.6 (1.0-97.1) [2.5-5.3]	2.3 (−1.9 to 6.6)	.29
Other, non-Hispanic	3.6 (1.2-26.5) [2.5-4.6]	−3.7 (−18.1 to 13.4)	.65
Maximum pain score at baseline^a^			
Overall (1-unit increase)	NA	1.3 (0.6 to 1.9)	.001
0	3.2 (1.0-31.2) [2.2-5.0]	NA	NA
1-3	3.0 (1.0-15.1) [2.2-4.7]	NA	NA
4-7	3.3 (1.0-51.6) [2.3-4.8]	NA	NA
8-10	4.0 (1.0-97.9) [2.8-6.4]	NA	NA
Total MED at baseline			
Overall (1-unit increase)	NA	2.4 (2.2 to 2.7)	<.001
0	3.0 (1.0-34.2) [2.1-4.5]	NA	NA
1-4	3.4 (1.0-60.5) [2.2-4.9]	NA	NA
5-8	3.6 (1.0-83.9) [2.6-5.2]	NA	NA
9-14	3.9 (1.0-51.6) [2.8-6.1]	NA	NA
≥15	5.0 (1.0-97.9) [3.2-8.7]	NA	NA
Organ failure at baseline			
No	3.6 (1.0-83.9) [2.5-5.5]	1 [Reference]	NA
Yes	8.7 (1.3-97.9) [4.8-22.4]	119.0 (98.4 to 141.7)	<.001
SIRS at baseline			
No	3.3 (1.0-60.5) [2.4-5.0]	1 [Reference]	NA
Yes	4.6 (1.0-97.9) [3.0-7.2]	24.2 (19.6 to 29.1)	<.001
Charlson Comorbidity Index score			
0	3.5 (1.0-97.9) [2.5-5.1]	1 [Reference]	NA
1-2	3.7 (1.0-97.1) [2.5-5.7]	3.3 (−0.8 to 7.5)	.12
≥3	4.2 (1.0-78.2) [2.8-6.4]	12.0 (5.9 to 18.4)	<.001
Missing	3.9 (1.1-81.3) [2.7-5.8]	7.0 (−0.4 to 15.0)	.07
Etiology of AP			
Gallstone related	3.9 (1.0-97.1) [2.8-5.8]	1 [Reference]	NA
Alcohol related	3.3 (1.0-97.9) [2.2-5.1]	−18.6 (−23.3 to −13.7)	<.001
Other	3.2 (1.0-83.9) [2.2-5.3]	−17.7 (−21.1 to −14.0)	<.001
Alcohol use disorder history			
Never	3.7 (1.0-97.1) [2.6-5.6]	1 [Reference]	NA
Ever	3.7 (1.0-97.9) [2.5-5.4]	1.8 (−3.3 to 7.2)	.49
Unknown	3.9 (1.0-60.5) [2.6-6.4]	11.3 (5.1 to 17.9)	<.001
Smoking history			
Never	3.7 (1.0-97.9) [2.6-5.5]	1 [Reference]	NA
Current	3.2 (1.0-47.0) [2.3-4.9]	−9.8 (−15.2 to −4.1)	<.001
Former	3.8 (1.0-97.1) [2.6-5.8]	−0.3 (−4.7 to 4.3)	.89
Missing or unknown	3.7 (1.0-60.5) [2.4-5.8]	−6.4 (−11.8 to −0.6)	.03
Medical center^b^			
K	3.8 (1.0-81.3) [2.7-6.0]	1 [Reference]	NA
A	3.7 (1.0-78.2) [2.6-5.3]	−5.9 (−12.3 to 1.1)	.10
B	3.7 (1.1-56.5) [2.7-5.2]	−0.9 (−7.7 to 6.3)	.80
C	3.6 (1.1-43.9) [2.3-5.1]	−11.3 (−17.7 to −4.4)	.002
D	3.4 (1.0-60.5) [2.2-5.1]	−6.5 (−13.4 to 0.9)	.08
E	3.9 (1.2-17.8) [2.4-5.8]	−6.7 (−18.7 to 7.0)	.32
F	3.5 (1.0-59.0) [2.6-5.4]	−1.5 (−9.2 to 6.8)	.71
G	3.7 (1.0-97.1) [2.7-5.8]	1.3 (−7.1 to 10.4)	.78
H	3.0 (1.0-24.1) [2.0-4.8]	−20.1 (−29.2 to −9.8)	<.001
I	3.0 (1.0-83.9) [2.0-5.0]	−17.7 (−23.8 to −11.2)	<.001
J	3.5 (1.0-97.9) [2.6-5.5]	−3.0 (−10.2 to 4.7)	.44
L	4.5 (1.0-78.2) [2.8-6.6]	8.6 (0.4 to 17.5)	.04
M	3.9 (1.1-67.6) [2.7-5.8]	−0.7 (−9.0 to 8.3)	.87
N	3.9 (1.0-57.0) [2.8-6.3]	11.1 (2.9 to 20.1)	<.001

Abbreviations: AP, acute pancreatitis; IQR, interquartile range; LOS, length of stay; MED, morphine equivalent dose; NA, not applicable; SIRS, systemic inflammatory response syndrome.

^a^ Baseline period is defined as the first 12 hours after the admission of index hospitalization for acute pancreatitis.

^b^ Medical centers are identified by anonymized letters.

### Persistent Opioid Use After Discharge

Among the initial cohort of 4307 patients, 286 (6.6%) were excluded owing to death, disenrollment from the health plan, or loss of prescription drug benefit within 180 days after discharge. Patient demographic, clinical, and institutional characteristics are presented by opioid dispensation status in the remaining 4021 patients in the eTable in the Supplement. Overall, 1680 patients (41.8%) were prescribed opioids only at discharge or within 14 days after discharge (nonpersistent opioid users), and 388 patients (9.6%) not only had opioids dispensed at discharge or within 2 weeks after discharge but also continued to receive opioids 90 to 180 days after discharge (persistent opioid users). Persistent opioid use was more common among patients who were aged 45 to 64 years, had a history of alcohol use disorder or tobacco use, or had increased LOS (eTable in the Supplement). In multivariable regression analysis, the odds of persistent opioid use increased by 2% for every 1-unit increase of baseline MED (odds ratio, 1.02; 95% CI, 1.00-1.04; *P* = .03) and 2% for every 1-unit increase of average MED per day during the entire hospital stay (odds ratio, 1.02; 95% CI, 1.01-1.03; *P* = .001) after adjusting for age, sex, race/ethnicity, maximum pain score at baseline, persistent organ failure during hospitalization (yes or no), persistent SIRS during hospitalization (yes or no), Charlson Comorbidity Index score, etiology of AP, smoking history, alcohol use disorder history, medical center, and alcohol and other substance use disorders (eTable in the Supplement).

## Discussion

We performed a broad assessment of opioid use in the treatment of patients with AP hospitalized within a US community-based integrated health care system. In addition to expected factors, such as intensity of pain and degree of systemic inflammation, we identified significant variation in opioid administration based on patient-related factors (eg, age, sex, race/ethnicity), etiology of AP, and medical center. Increased administration of opioids at was an independent risk factor for longer duration of hospitalization. Nearly 10% of patients went on to receive new persistent opioid prescriptions up to 6 months after hospitalization.

Acute pancreatitis is a common cause of hospitalization, with more than 250 000 annual admissions across the United States,^2^ and AP ranks as one of the most common causes of hospitalization related to a digestive illness.^1,3^ Intravenous opioids have been administered for relief of acute pain in patients hospitalized for AP for decades. However, the use of opioids in the routine care of patients with AP has received little attention and, to our knowledge, is not specifically addressed in clinical practice guidelines in AP.^4,5,6^ A 2013 Cochrane review^15^ and a 2013 independent systematic review^16^ of previous trials comparing opioid- vs nonopioid-based analgesic regimens found little evidence to support superiority of any approach to analgesia in patients with AP. In the present study, most (80%) patients received initial analgesia with opioids. In the absence of clear guidelines on the appropriate use of opioid-based analgesia, it is not surprising that there was significant variation in opioid prescribing patterns. However, after controlling for pain, systemic inflammation, and etiology of AP, patient-related factors, such as non-Hispanic white race/ethnicity, and medical center of treatment emerged as independent risk factors for increased receipt of opioids. These findings are consistent with previous data that indicate significant variation in opioid prescriptions in emergency departments based on race/ethnicity^17^ and on individual health care institution.^18^

Increased administration of opioids during the baseline period of hospitalization was associated with longer duration of hospital stay. There are several possible explanations for this finding. While it is possible that refractory pain could be a marker for more extensive or severe disease, it is also conceivable that excessive use of opioids may contribute to delayed restoration of gut function, particularly gastric motility,^19^ in patients with AP. In addition, data from a 2018 experimental study^20^ using animal models suggest a potential direct deleterious effect of opioids on exacerbating inflammation and inhibiting tissue recovery in the setting of AP.

Although 51% of patients continued to receive opioids at the time of discharge, relatively few patients (<10%) went on to become persistent opioid users within 90 to 180 days after discharge. Although readily comparable data in AP are lacking, previous estimates of persistent opioid use among patients recovering from surgical procedures have ranged from 5% to 6%.^11,21^ The increased rate of new persistent opioid use observed in the present study could be associated with development of chronic pancreatitis and is consistent with previous estimates of approximately 6% to 12% of patients progressing to chronic pancreatitis after an initial episode of AP.^22,23^

The findings have several relevant implications. The variation in opioid prescribing patterns based on patient-related factors reinforces the need to develop evidence-based recommendations for optimal approaches to analgesia in patients with AP. Several additional steps are needed to help develop treatment strategies that address this challenge. First, there is a need to better understand the underlying patterns of pain in AP and to assess physician and patient perspectives regarding analgesia in this setting. Second, development of care pathways that incorporate elements of enhanced recovery approaches after surgical procedures may offer opportunities to reduce opioid use while expediting recovery in patients with AP.^24^ Additionally, our finding that nearly 10% of patients went on to persistent opioid use suggests that early identification of patients at risk for persistent opioid use and treatment with nonopioid regimens after discharge may provide an opportunity to reduce long-term opioid dependency.

### Limitations and Strengths

There were several limitations of this study. We used the total dose of opioids during the baseline period (initial 12 hours) as a surrogate measure of overall prescribing patterns. This approach helped focus the analysis within a defined period of hospitalization during which all patients in the study cohort were at equal risk of exposure (receipt of opioids). However, this approach limited our ability to account for additional downstream events when assessing the potential association of opioid use with overall LOS. When evaluating risk factors for persistent opioid use after discharge, both MED at baseline and mean daily MED was independently associated with increased risk of long-term use of opioids. In our analysis, we used maximum recorded pain score on a numeric rating scale as a measure of pain intensity during the initial phase of hospitalization. This approach did not account for the duration of pain, nor were we able to assess the response to initial treatment. Additionally, the study included data collected from 2008 to 2015 and does not capture the potential effects of more recent initiatives to curb administration of opioids in inpatient settings.^24,25^

Despite these limitations, our study has several important strengths. As a multihospital, community-based care system, we were able to analyze prescribing patterns for opioids across multiple institutions caring for a racially/ethnically diverse patient population. In addition, as an integrated health care system, we were able to reliably identify opioid prescriptions in the study cohort after discharge. Finally, the relatively large sample size and detailed clinical information available through the electronic health record system enabled us to conduct a broad assessment of both inpatient and outpatient use of opioids for patients with AP.

## Conclusions

In this community-based cohort study of patients hospitalized for AP, we determined that 80% of patients received opioids during the initial stages of hospitalization. Opioid administration varied according to patient factors, including pain level recorded at baseline, systemic inflammation, etiology of AP, sex, and race/ethnicity. Opioid administration also varied by facility. Increased administration of opioids during the initial 12 hours of hospitalization was an independent risk factor associated with longer duration of hospitalization. While more than 50% of patients continued to receive opioids at discharge, almost 10% of patients continued to receive persistent opioids up to 6 months after discharge. Further evidence-based recommendations for nonopioid-based analgesia are urgently needed to help reduce disparities in and potentially enhance outcomes for patients with AP.

## Notes

Supplement. **eTable.** Frequency of Persistent Opioid Use and Adjusted Odds Ratio and 95% CI for Persistent Opioid Use
