# Supplementary material for: Factors Associated With Opioid Use in Patients Hospitalized for Acute Pancreatitis
Source: JAMA Netw Open. 2019 Apr 12;2(4):e191827. doi: 10.1001/jamanetworkopen.2019.1827 (PMC6481601; doi:10.1001/jamanetworkopen.2019.1827)
Supplement: Supplement. — eTable. Frequency of Persistent Opioid Use and Adjusted Odds Ratio and 95% CI for Persistent Opioid Use [file jamanetwopen-2-e191827-s001.pdf]

Supplementary Online Content

Wu BU, Butler RK, Chen W. Factors associated with opioid use in patients hospitalized for acute pancreatitis. *JAMA Netw Open*. 2019;2(4):e191827. doi:10.1001/jamanetworkopen.2019.1827

eTable. Frequency of Persistent Opioid Use and Adjusted Odds Ratio and 95% CI for Persistent Opioid Use

This supplementary material has been provided by the authors to give readers additional information about their work.

© 2019 Wu BU et al. *JAMA Network Open.*

eTable. Frequency of Persistent Opioid Use and Adjusted Odds Ratio and 95% CI for Persistent Opioid Use

Patient Characteristic	Frequency of Persistent Opioid Use N (%)	Adjusted OR (95% CI)	P-value
Age (years)		(ref)	(ref)
18 – 44	100 (10.3)	1.10 (0.84, 1.44)	0.047
45 – 64	171 (10.9)	1.05 (0.75, 1.47)	0.128
65 – 84	101 (8.5)	0.45 (0.19, 1.08)	0.049
85+	6 (3.2)		
Sex		1.21 (0.96, 1.53)	0.104
Female	205 (9.8)	(ref)	(ref)
Male	183 (9.5)		
Race/ ethnicity		0.98 (0.62, 1.54)	0.542
Asian, non-Hispanic	27 (8.2)	1.27 (0.86, 1.87)	0.433
Black, non-Hispanic	46 (12.0)	0.97 (0.74, 1.26)	0.312
Hispanic	158 (9.4)	1.37 (0.52, 3.64) (ref)	0.583
Other, non-Hispanic	5 (11.4)		(ref)
White, non-Hispanic	152 (9.6)		
Maximum pain score (baseline)		1.05 (1.01, 1.10) for every one unit increase of maximum pain score	0.030
0	13 (3.5)		
1-3	16 (6.4)		
4-7	110 (8.6)		
8-10	249 (11.8)		
Baseline MED[b]		1.02 (1.00, 1.04) for every one unit increase of baseline MED[a]	0.032
0	39 (4.9)		
1-4	79 (8.4)		
5-8	67 (8.1)		
9-14	83 (11.5)		
15+	120 (16.2)		
Average MED[b] per day during the entire hospital stay	14 (3.6)	1.02 (1.01, 1.03) for every one unit increase of average MED[a]	0.001
0	87 (6.5)		
1-4	95 (10.3)		
5-10	102 (12.1)		
10-20	90 (17.1)		
20+			
Persistent organ failure during hospitalization		(ref)	(ref)
No	377 (9.5)	1.42 (0.68, 2.96)	0.349
Yes	11 (15.7)		
Persistent SIRS during hospitalization		(ref)	(ref)
No	335 (9.2)	1.17 (0.82, 1.67)	0.394
Yes	53 (14.0)		
Charlson Comorbidity Index		(ref)	(ref)
0	155 (9.1)	1.42 (1.11, 1.82)	0.003
1 to 2	164 (11.5)	1.00 (0.69, 1.46)	0.682
3 or More	48 (7.6)	0.89 (0.54, 1.46)	0.355
Missing	21 (8.3)		
Etiology			
Gallstone Disorders	217 (9.8)	(ref)	(ref)
Alcohol-Related	80 (11.7)	0.80 (0.57, 1.13)	0.507
Other	91 (8.1)	0.83 (0.62, 1.10)	0.639
Alcohol use history			0.096
Ever	162 (11.7)	1.22 (0.90, 1.67) (ref)	(ref)
Never	187 (8.9)	0.85 (0.57, 1.27)	0.194
Unknown	39 (7.3)		
Smoking history		1.34 (0.88, 2.05)	0.193
Current	56 (14.6)	1.13 (0.85, 1.50) (ref)	0.812
Former	93 (10.0)	0.96 (0.64, 1.43)	(ref)
Never	200 (8.9)		0.370
Missing/Unknown	39 (8.5)		
Medical center			
A	44 (11.1)	1.32 (0.86, 2.03)	0.116
B	36 (8.9)	0.93 (0.59, 1.46)	0.611
C	51 (15.9)	1.70 (1.12, 2.58)	0.001
D	21 (6.8)	0.79 (0.46, 1.34)	0.253
E	11 (16.2)	1.58 (0.77, 3.26)	0.168
F	26 (9.4)	1.11 (0.66, 1.86)	0.690
G	16 (7.7)	0.82 (0.45, 1.47)	0.392
H	11 (12.1)	1.22 (0.60, 2.46)	0.556
I	31 (10.5)	1.02 (0.64, 1.64)	0.967
J	25 (8.9)	0.89 (0.54, 1.47)	0.517

© 2019 Wu BU et al. *JAMA Network Open.*

K	59 (9.8)	(ref)	(ref)
L	24 (8.6)	0.84 (0.50, 1.40)	0.369
M	17 (7.4)	0.78 (0.43, 1.41)	0.305
N	19 (6.6)	0.73 (0.42, 1.26)	0.156
Alcohol and other substance use disorders[c]			(ref)
No	312 (9.0)	(ref)	0.625
Yes	76 (13.6)	1.10 (0.76, 1.58)	

© 2019 Wu BU et al. *JAMA Network Open.*
